# Commentary: Police and Suicide Prevention

**DOI:** 10.3389/fpubh.2016.00119

**Published:** 2016-06-08

**Authors:** Leo Sher

**Affiliations:** ^1^James J. Peters Veterans’ Administration Medical Center, Bronx, NY, USA; ^2^Icahn School of Medicine at Mount Sinai, New York, NY, USA

**Keywords:** police, suicide, drugs, antisocial behavior

I read with interest an important article, “Police and suicide prevention” that was recently published in *Crisis* (1). The authors developed and evaluated training in suicide awareness and prevention for frontline police officers. Areas covered in the training module included the role of police officers in suicide prevention, the main characteristics of individuals who engage in suicidal and self-harm acts, how to approach and question individuals suspected to be at risk of suicide, how to ascertain the level of risk of individuals suspected to be suicidal or expressing suicidal ideation, how to refer individuals at risk of suicide to the services most appropriate for their specific needs and level of risk, and other issues. All officers who participated in the training program were asked to complete a questionnaire before, immediately after, and 6 months after undertaking training, to measure changes in knowledge, confidence, and attitudes, regarding suicide prevention. The authors concluded that training in suicide prevention appears to have been well received and to have had a beneficial impact on officers’ attitudes, confidence, and knowledge. The results of this study show that police may play a role in suicide prevention. The authors also astutely noted that providing training with regard to suicidality may help the officers themselves in terms of increasing awareness and recognition of mental health issues that might affect them personally, their colleagues, or their family members. This is important because substantial job-related stressors and exposures lead to increased risk for psychiatric issues, including suicidal behavior among police officers (2–4). It should be noted that studies related to crisis intervention and suicide prevention training for police officers have been conducted in several countries (5–12).

Police may play a role in suicide prevention not only because police officers may prevent suicides when they interact with suicidal individuals. Police may contribute to suicide prevention indirectly.

The use of illicit drugs is associated with suicide (13). For example, a number of cross-sectional or retrospective studies have found an association between cannabis use and suicidal ideation, suicide attempts, or completed suicide (14–20). In one of these studies, a research group in Brisbane, Australia examined same sex twin pairs discordant for cannabis dependence to determine relationships between cannabis use and major depressive disorder, suicidal behavior, and suicidal ideations (14). Cannabis-dependent individuals had odds of suicide attempt or ideation that was 2.5–2.9 times higher than their non-cannabis-dependent twin. In another example, a research report suggests that heroin users are 14 times more likely than peers to die from suicide (21). Deaths among heroin users attributed to suicide range from 3 to 35% (21). A study in Rome, Italy has reported excess suicide mortality of 6.3 times among heroin users to that expected among matched peers (22). Police and other law enforcement agencies in many countries work hard to fight drug crimes. For example, in the U.S., there is a drug arrest every 19 seconds (23). When police fight drug trafficking and distribution, they decrease the availability of drugs, which may reduce suicides.

Antisocial behavior is also associated with suicide (24–28). For example, it has been reported that “Studies have revealed antisocial personality disorder or criminal behavior to be a predictor of subsequent suicide attempts” (28). Police Community Outreach Programs and other similar projects may reduce antisocial behavior, especially among young people (21–32). Consequently, this may reduce suicides. In summary, good police work may decrease suicide rates, directly and indirectly.

## Author Contributions

The author confirms being the sole contributor of this work and approved it for publication.

## Conflict of Interest Statement

The author declares that the research was conducted in the absence of any commercial or financial relationships that could be construed as a potential conflict of interest.
